# Structure and RAF family kinase isoform selectivity of type II RAF inhibitors tovorafenib and naporafenib

**DOI:** 10.1016/j.jbc.2023.104634

**Published:** 2023-03-22

**Authors:** Emre Tkacik, Kunhua Li, Gonzalo Gonzalez-Del Pino, Byung Hak Ha, Javier Vinals, Eunyoung Park, Tyler S. Beyett, Michael J. Eck

**Affiliations:** 1 Department of Cancer Biology, Dana-Farber Cancer Institute, Boston, Massachusetts, USA; 2 Department of Biological Chemistry and Molecular Pharmacology, Harvard Medical School, Boston, Massachusetts, USA

**Keywords:** RAF kinase, fluorescence resonance energy transfer (FRET), x-ray crystallography, inhibitor mechanism, cooperativity, drug action

## Abstract

Upon activation by RAS, RAF family kinases initiate signaling through the MAP kinase cascade to control cell growth, proliferation, and differentiation. Among RAF isoforms (ARAF, BRAF, and CRAF), oncogenic mutations are by far most frequent in BRAF. The BRAF^V600E^ mutation drives more than half of all malignant melanoma and is also found in many other cancers. Selective inhibitors of BRAF^V600E^ (vemurafenib, dabrafenib, encorafenib) are used clinically for these indications, but they are not effective inhibitors in the context of oncogenic RAS, which drives dimerization and activation of RAF, nor for malignancies driven by aberrantly dimerized truncation/fusion variants of BRAF. By contrast, a number of “type II” RAF inhibitors have been developed as potent inhibitors of RAF dimers. Here, we compare potency of type II inhibitors tovorafenib (TAK-580) and naporafenib (LHX254) in biochemical assays against the three RAF isoforms and describe crystal structures of both compounds in complex with BRAF. We find that tovorafenib and naporafenib are most potent against CRAF but markedly less potent against ARAF. Crystal structures of both compounds with BRAF^V600E^ or WT BRAF reveal the details of their molecular interactions, including the expected type II–binding mode, with full occupancy of both subunits of the BRAF dimer. Our findings have important clinical ramifications. Type II RAF inhibitors are generally regarded as pan-RAF inhibitors, but our studies of these two agents, together with recent work with type II inhibitors belvarafenib and naporafenib, indicate that relative sparing of ARAF may be a property of multiple drugs of this class.

RAF kinases (ARAF, BRAF, and CRAF/RAF1) are key effectors of RAS that initiate signaling through the MAPK signaling cascade (1, 2, 3). Upon activation by RAS, RAFs phosphorylate their sole substrates MEK1/2, which then phosphorylate ERK1/2 (4, 5). Through their role at the apex of this pathway, they regulate multiple cellular processes, including growth and proliferation (6). RAF activity is tightly regulated by intramolecular interactions, phosphorylation, and binding partners (7). In the absence of RAS activation, RAF proteins exist in an autoinhibited state as a complex with MEK and a 14-3-3 dimer (6). Structural studies of BRAF show how the 14-3-3 domain binds phosphoserine motifs that flank the kinase domain, restraining the kinase and its N-terminal cysteine-rich domain in a manner that precludes dimerization of the kinase (7, 8). By contrast, in the active state, the 14-3-3 dimer rearranges to bind the C-terminal phosphoserine sites in two RAF molecules, driving and stabilizing formation of the “back to back” dimerization of the kinase domain that is the trigger for catalytic activation (7, 8, 9, 10, 11, 12, 13). In both active and inactive states, the BRAF kinase domain coordinates MEK in a “face-to-face” orientation (7, 8, 14). Phosphorylation of the MEK activation loop on serines 218 and 222 by RAF activates MEK and results in its release from RAF (6, 15).

Oncogenic mutations can short-circuit this normal regulatory apparatus by manipulating the mechanism of RAF dimerization or by circumventing this requirement entirely (16, 17). Among the three RAF isoforms, BRAF is by far the most commonly mutated (18). Oncogenic mutations in BRAF can be divided into three distinct classes: monomerically activating mutations, kinase-impaired transactivating mutations, and RAS-independent dimerizing mutations (17, 19, 20, 21). The most prevalent BRAF mutation, BRAF^V600E^, falls into the first of these classes and removes BRAF’s dependance on dimerization and RAS activation for its kinase activity (22). The BRAF^V600E^ mutation is responsible for the majority of all malignant melanomas and papillary thyroid carcinomas, and is found in many other cancers as well (23).

Several selective BRAF^V600E^ inhibitors are used to treat malignant melanoma and certain other BRAF^V600E^-mutated cancers, typically in combination with a partner MEK inhibitor (6, 24, 25). Addition of a MEK inhibitor blunts paradoxical activation of the MAPK pathway, which is a major liability of current RAF inhibitors, and also improves efficacy (6, 24). Left unchecked, paradoxical activation can lead to the development of secondary skin malignancies, such as squamous cell carcinomas and keratoacanthomas (26, 27). Approved RAF/MEK inhibitor combinations include dabrafenib/trametinib, vemurafenib/cobimetinib, and encorafenib/binimetinib (28). The BRAF^V600E^-selectivity of these RAF inhibitors stems from their binding mode and from the fact that the BRAF^V600E^ mutant is active and signals as a monomer (22). Binding of these inhibitors requires an outward displacement of the C-helix that is only readily accessible in the monomer state (10, 29, 30). This binding mode is referred to as “Type 1.5” to distinguish it from type I inhibitors, which also occupy the ATP site, but do not require the C-helix-out conformation (31). Dimerization of the RAF kinase domain forces the inward, active position of the C-helix and thereby antagonizes binding of type 1.5 inhibitors (10, 29). For this reason, these agents are ineffective against RAF dimers and are referred to as RAF-monomer inhibitors (32, 33, 34).

A number of agents that potently inhibit RAF dimers have also been developed. Most of these drugs exhibit a “Type II” binding mode, which is characterized by a “DGF-out” conformation of the kinase (31, 35). The DFG-motif is a conserved three-residue segment (Asp-Phe-Gly) that lies at the N-terminus of the kinase activation loop. Type II inhibitors bind (or induce) a conformation created by a crankshaft-like flip of the DFG segment that reorients the phenylalanine residue toward the ATP site (35). Inhibitors with a type II–binding mode extend from the ATP site to insert a hydrophobic group in the site vacated by the DFG phenylalanine (35). Tovorafenib (also referred to as DAY101, TAK-580) and naporafenib (LXH254) are among the type II inhibitors currently in clinical development (36, 37). In murine models of pediatric low-grade glioma (PLGA) and in human PLGA cells, tovorafenib has been shown to potently inhibit KIAA1549:BRAF, a truncation/fusion variant of BRAF that lacks key regulatory regions in the kinase N-terminus and as a result is constitutively dimerized (37, 38, 39, 40). In addition to being potent against KIAA1549:BRAF, tovorafenib is brain-penetrant, and as such, it is being evaluated in PLGA, a brain tumor that is frequently driven by the KIAA1549:BRAF fusion (37, 41).

Although type II RAF inhibitors are generally considered to be pan-RAF inhibitors, effective against each of the RAF isoforms, both belvarafenib and naporafenib have recently been shown to inhibit ARAF only weakly (32, 36, 42). To better understand the isoform selectivity of tovorafenib and naporafenib, we measured their *in vitro* potencies against purified dimers of each RAF isoform and against BRAF^V600E^ monomers. We also determined cocrystal structures of tovorafenib bound to WT BRAF and BRAF^V600E^ kinase domains and of naporafenib bound to WT BRAF. We find that like naporafenib, tovorafenib is a poor inhibitor of ARAF. Furthermore, our cocrystal structures provide a molecular basis for understanding the inhibition of BRAF dimers by these agents.

## Results

### Preparation of active, 14-3-3–bound RAF dimers for inhibitor characterization

**Figure 1:**
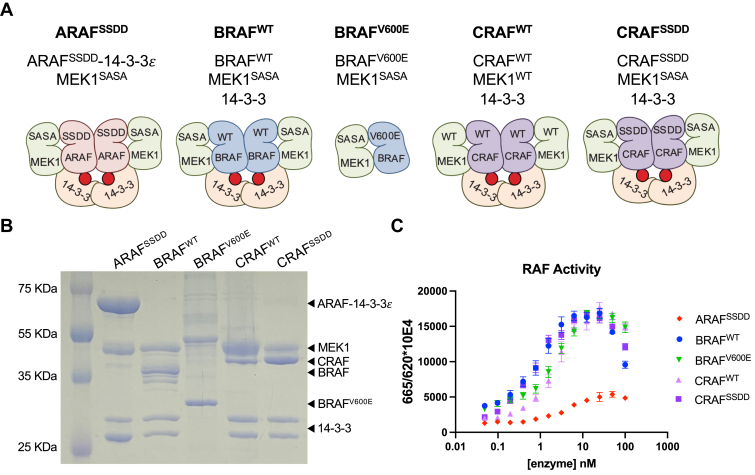
**RAF protein constructs and their activity.***A*, schematic of RAF complexes studied here. For simplicity, we refer to these preparations as ARAF^SSDD^, BRAF^WT^, BRAF^V600E^ CRAF^WT^, and CRAF^SSDD^, as indicated. *B*, coomassie-stained SDS-PAGE gel of purified RAF preparations. *C*, activity of purified RAF complexes as assayed by TR-FRET. Ratio of emission at 665/620 nm is plotted for increasing concentrations of each RAF preparation. TR-FRET, time-resolved FRET.

Most prior biochemical studies of RAF inhibitors have employed either the truncated kinase domain of the RAF isoform of interest or the full-length protein (42). In our experience, full-length RAF proteins are highly prone to aggregation and usually yield a mixture of monomeric and dimeric species. In order to obtain each of the RAF isoforms in a more homogeneous dimeric state, we designed RAF constructs that include the kinase domain and C-terminal 14-3-3–binding motif for expression in the baculovirus/insect cell system. We coexpressed each with a MEK1 variant with alanine mutations in its activation loop’s phosphorylation sites (S218A/S222A, we refer to this construct as MEK1^SASA^). Coexpression with MEK dramatically improves recovery and stability of RAF. Incorporation of the activation loop substitutions in MEK stabilizes the complex by preventing its phosphorylation and release from RAF. The BRAF–MEK complexes copurify with an endogenous insect cell 14-3-3 dimer, thus forming an active back-to-back BRAF dimer. We also purified a BRAF^V600E^ construct, again coexpressed with MEK^SASA^, but lacking both 14-3-3–binding motifs, resulting in the formation of a monomeric BRAF^V600E^ complex bound to MEK^SASA^ that is constitutively active due to the V600E mutation (MEK1^SASA^:BRAF^V600E^). These complexes are depicted schematically in Figure 1*A*, and an SDS-PAGE gel of the purified proteins is shown in Figure 1*B*.

For CRAF, we prepared both the WT sequence and a mutant construct with Y340D/Y341D mutations in a putative regulatory phosphorylation site at the N-terminus of the kinase domain. Mutation of this “SSYY” sequence (residues 338–341) in CRAF to “SSDD” as found in BRAF has previously been shown to yield a more active CRAF protein (43). As with BRAF, we coexpressed the CRAF^SSDD^ construct with MEK^SASA^. For reasons we do not understand, the corresponding WT CRAF construct tended to aggregate when coexpressed with MEK1^SASA^ but not when coexpressed and purified with WT MEK1 (MEK1^WT^), which we therefore used to prepare the WT CRAF complex. As with BRAF, both WT and mutant CRAF constructs bind endogenous 14-3-3 proteins and form active dimer complexes (Fig. 1, *A* and *B*).

This strategy was not successful with ARAF, as we obtained little 14-3-3–bound active dimer. As an alternate approach, we designed a single-chain chimera in which we fused 14-3-3ε to the C-terminus of the ARAF kinase construct. As with our mutant CRAF construct, we prepared the ARAF^SSDD^ variant in which the native “SGYY” sequence in ARAF (residues 299–302) was mutated to match the BRAF motif. Coexpression of this chimeric construct with MEK1^SASA^ yielded active ARAF dimer (MEK1^SASA^:ARAF^SSDD^-14-3-3 ε), albeit with some heterogeneity, apparently due to the formation of heterodimers with free insect cell 14-3-3 at the expense of complete homodimerization of fused/chimeric 14-3-3 domains (Fig. 1*B*).

We employed a well-established time-resolved FRET (TR-FRET) assay to measure phosphorylation of the MEK activation loop (S218/S222) by these RAF complexes (44). This assay employs biotinylated WT MEK1 as a substrate. Phosphorylation of the MEK1 activation loop (on residues S218/S222) is detected with an anti-phospho MEK1/2 antibody coupled to Eu^3+^ (Cisbio) as the FRET donor and an XL665-streptavidin conjugate as a FRET acceptor. All the RAF preparations described above are active, although the ARAF^SSDD^ complex is considerably less active the BRAF and CRAF complexes (Fig. 1*C*). In consensus with previous reports, CRAF^SSDD^ is more active than CRAF^WT^. Although the MEK1^SASA^ component of these RAF complexes is expected to act as a competitive inhibitor of phosphorylation of the MEK1^WT^ substrate, the effect is negligible when the concentration of the RAF–MEK1^SASA^ enzyme complex is much less than that of the MEK1^WT^ substrate. We do note a decrease in observed activity at the highest enzyme concentrations tested (50–100 nM), which likely stems from this effect as the concentration of MEK1^SASA^ begins to approach that of the MEK1^WT^ substrate (250 nM).

### Potency of tovorafenib and naporafenib across RAF isoforms

**Figure 2:**
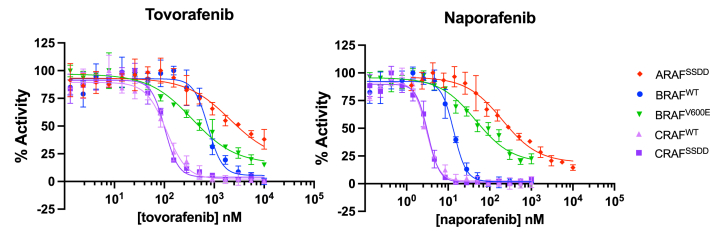
**Representative concentration-response curves of tovorafenib and naporafenib generated with the purified RAF complexes described in**Figure 1**.** ARAF^SSDD^ dimer and BRAF^V600E^ monomer dose-response curves have a standard Hill slopes (−1.0) but BRAF^WT^, CRAF^WT^, and CRAF^SSDD^ dimer curves have nonstandard Hill slopes less than −1.0, indicating that these dimer RAF complexes are inhibited with positive cooperativity. Data are plotted as mean ± SD from one independent experiment performed in triplicate (n = 3).

To better understand the RAF selectivity of tovorafenib and naporafenib, we measured their inhibition of the RAF complexes described above using our adapted TR-FRET assay. BRAF^WT^, BRAF^V600E^, and CRAF^SSDD^ were assayed at a concentration of 1 nM, while CRAF^WT^ and ARAF^SSDD^ were assayed at 4 nM and 10 nM, respectively, due to their lower enzymatic activities. An ATP concentration of 200 μM was used for all assays, and the WT MEK1 substrate concentration was 250 nM. Measured IC_50_ values and calculated K_i_ values are provided in Table 1, and representative concentration-response curves from which they were derived are shown in Figure 2.

**Table 1:** Enzymatic IC_50_, hill slope, and K_i_ values for ARAF, BRAF, and CRAF proteins^a^

	Tovorafenib			Naporafenib		
	IC_50_ (nM)	Hill slope	K_i_ (nM)	IC_50_ (nM)	Hill slope	K_i_ (nM)
ARAF^SSDD^	>3000	−1.12 ± 0.42	>40	414 ± 186	−0.78 ± 0.05	5.71 ± 2.97
BRAF^WT^	633 ± 160	−2.89 ± 0.19	6.13 ± 5.33	13.4 ± 0.30	−2.73 ± 0.23	0.13 ± 0.08
BRAF^V600E^	495 ± 117	−0.75 ± 0.01	20.5 ± 12.4	49.2 ± 3.00	−0.96 ± 0.09	2.04 ± 0.83
CRAF^WT^	94.2 ± 13.2	−1.55 ± 0.43	1.03 ± 0.99	3.66 ± 0.65	−2.57 ± 0.65	0.04 ± 0.03
CRAF^SSDD^	84.5 ± 15.8	−3.18 ± 0.08	0.21 ± 0.06	3.64 ± 0.36	−3.03 ± 0.73	0.01 ± 0.001

a Inhibitor titrations were performed in triplicate in three independent experiments, and IC_50_ values are reported as mean ± SD. K_i_ values were calculated from the IC_50_ values using the Cheng-Prusoff equation (K_i_ = IC_50_/1+ [ATP]/K_m-ATP_) and are reported as mean ± error due to variance in the experimentally determined IC_50_ and K_m-ATP_ values.

Both tovorafenib and naporafenib were most potent as inhibitors of CRAF, with IC_50_ values of 94.2 nM and 3.7 nM, respectively, against the WT CRAF kinase. Potency against the CRAF^SSDD^ mutant was essentially the same as for CRAF^WT^ (Table 1). Both agents exhibited intermediate potency against BRAF^WT^ and BRAF^V600E^ (633 nM for tovorafenib and 13.4 nM for naporafenib on BRAF^WT^) and were much weaker inhibitors of ARAF^SSDD^. Tovorafenib did not completely inhibit ARAF^SSDD^ even at 10 μM, the highest inhibitor concentration we could achieve in this assay. While tovorafenib and naporafenib share potency trends across the RAF isoforms, naporafenib is consistently more potent than tovorafenib against each enzyme by at least an order of magnitude. A prior study of naporafenib activity against purified ARAF, BRAF, and CRAF reported relative potencies similar to those we observe but with markedly lower IC_50_ values (0.07 nM for CRAF) (42). Reaction conditions were not provided for this study, precluding meaningful comparison with our results.

The very steep concentration-response curves for tovorafenib and naporafenib against CRAF and WT BRAF suggest positive cooperativity of inhibition of these RAF dimers (Fig. 2). Fitting of these curves with a four-parameter model to allow for a variable Hill slope resulted in Hill slopes ranging from −2.6 to −3.2 (Table 1). These values indicate that tovorafenib and naporafenib inhibit BRAF and CRAF dimers with marked positive cooperativity; that is, that binding of inhibitor to the active site of one protomer increases the affinity for inhibitor binding to the second protomer in the RAF dimer. We did not observe this effect with either ARAF^SSDD^ or with BRAF^V600E^, which is monomeric in this assay (Table 1).

### Structures of BRAF in complex with tovorafenib and naporafenib

**Figure 3:**
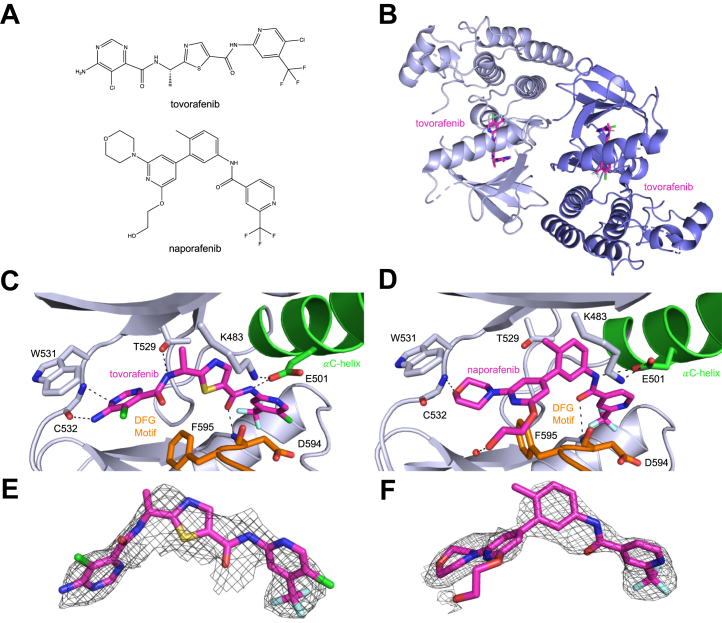
**Crystal structures of BRAF with tovorafenib and naporafenib.***A*, chemical structures of tovorafenib and naporafenib. *B*, the asymmetric unit of the BRAF^V600E^ structure contains a back-to-back BRAF^V600E^ dimer bound to tovorafenib, with the inhibitor bound in each protomer of the dimer. *C*, detailed view of tovorafenib in complex with BRAF^V600E^. Hydrogen bonds are depicted as *dashed lines*. The DFG-motif is colored *orange* and the αC-helix is shown in *green*. Tovorafenib spans the nucleotide-binding site, and the kinase adopts a DFG-out, αC-helix-in conformation. *D*, detailed view of naporafenib in complex with BRAF^WT^, colored as in panel *C*. *E* and *F*, simulated annealing composite omit electron density maps for tovorafenib (*E*) and naporafenib (*F*) in complex with BRAF^V600E^ and BRAF^WT^, respectively. Maps represent 2F_o_-F_c_ density contoured at 1σ.

For structural studies, we employed a previously described BRAF kinase domain construct containing 14 surface mutations that enable soluble expression in *Escherichia coli* and also facilitate crystallization (30, 45). Though not suitable for enzyme kinetic studies, this construct has been widely employed for crystallization of BRAF with inhibitors (45). We determined cocrystal structures with tovorafenib and naporafenib using this construct. For tovorafenib, we also determined a structure with BRAF containing the additional V600E oncogenic mutation. The structures were determined at resolutions ranging from 2.75 Å to 3.5 Å, with refinement statistics appropriate to their respective resolutions (Table S1, Supporting information). The naporafenib structure and both tovorafenib structures contain two copies of the kinase in their asymmetric unit, arranged in the back-to-back configuration typical of the active BRAF dimer. In all three structures, both protomers of the dimer contain clear inhibitor density, revealing a characteristic type II inhibitor–binding mode, with the DFG segment flipped into the DFG-out orientation (Figs. 3, *C*–*F* and S1*A*, Supporting information).

In the BRAF^V600E^ structure with tovorafenib, the inhibitor binds with its bisubstituted pyrimidine ring in the adenine-binding region of the ATP site, where it forms two hydrogen bonds to the kinase hinge (with the backbone amide and carbonyl groups of C532, Fig. 3*C*). The amide nitrogen on the left-hand side of the molecule hydrogen bonds with T529, and the central thiazole ring of the inhibitor is positioned between T529 and the active site lysine (K483). The adjacent carbonyl oxygen hydrogen bonds with the backbone amide of D594 in the DFG-motif, and the right-hand side amide nitrogen hydrogen bonds with E501 in the C-helix. Finally, the trifluoromethyl-substituted pyridine ring binds in the hydrophobic pocket vacated by the DFG phenylalanine (F595), with the trifluoromethyl group oriented toward the back of the pocket (Fig. 3*C*). An essentially identical binding mode is observed in the WT BRAF structure with tovorafenib (Fig. S1*A*, Supporting information). Density for the activation loops in both tovorafenib structures is not resolved between residues 599 to 613, which includes the site of the V600E mutation.

In the naporafenib crystal structure, the compound binds with its central substituted phenyl ring in the space between T529 and K483 and analogous to tovorafenib, with its trifluoromethyl pyridyl moiety tucked into the hydrophobic pocket opened by the flip of the DFG segment (Fig. 3*D*). On the other side of the molecule, the morpholine moiety forms a hydrogen bond with the C532 backbone amide in the kinase hinge (Fig. 3*D*). As with tovorafenib and most other type II inhibitors, the amide group hydrogen bonds with E501 in the C-helix and with the amide nitrogen of D594 (Fig. 3*D*). Overall, the binding mode of naporafenib in the present structure is closely similar to that observed in a prior structure with this compound, except that the hydroxyethyl group extends toward solvent and forms a hydrogen bond with a water molecule rather than turning to hydrogen bond with the carbonyl of F595 in the DFG-motif as in the prior structure (36).

These structures also reveal a potential structural basis for the observed cooperativity of inhibition by tovorafenib and naporafenib. The DFG-flip induced by these inhibitors is remote from the dimer interface, and overall, the dimer interface appears to be unperturbed. However, there is a small but significant change in the relative orientation of the kinase N- and C-lobes when bound to these agents, as compared with available structures of the active dimer in the absence of any inhibitor (Fig. S2, *A* and *B*, Supporting information). This difference in orientation is not due to crystal packing, as it is observed across multiple molecules in differing crystal packing environments. Thus, it seems likely that cooperativity is mediated by this change, communicated through the back-to-back dimer, which involves extensive contacts with both lobes of the kinase.

Interestingly, for one subunit of the dimer in the tovorafenib BRAF^WT^ structure, we observe formation of a domain-swapped dimer with an adjacent molecule in the crystal lattice (related by a crystallographic 2-fold axis of symmetry). The swapped region includes the αEF helix and flanking regions (residues 610–630) and appears to be facilitated by the unstructured activation loop, which leads into it (Fig. S1*B*, Supporting information). Similar domain-swaps, sometimes referred to as ‘activation segment exchange’, have been observed in a small number of other kinases (46, 47, 48, 49, 50, 51, 52, 53, 54) and was recently described in a BRAF structure with a novel inhibitor where it was thought to be ligand-induced (55). Our review of BRAF structures in the Protein Data Bank reveal a number of prior structures that exhibit this subdomain swap (including entries 5ITA, 4XV2, and 4CQE) (56, 57, 58), although the domain swap was not modeled as such in the deposited coordinates (Fig. S3, Supporting information). In BRAF, this effect appears to be an artifact of crystallization with the surface-mutagenized kinase, as all of the structures that exhibit the swap were determined with this approach.

## Discussion

Despite their distinct chemical structures, tovorafenib and naporafenib exhibit similar inhibitory properties *in vitro*; both inhibit BRAF and CRAF dimers with positive cooperativity and both are markedly less potent against ARAF (Table 1). Our cocrystal structures of these compounds with BRAF reveal the DFG-out–binding mode that is characteristic of type II inhibitors and also reveal a modest opening of the relative orientation of the N- and C-lobes of the kinase that may underlie cooperative inhibition of these RAF dimers. The structures do not, however, provide insight into the lack of potency against ARAF. The residues that contact the inhibitors are identically conserved across the three RAF isoforms, and at least three structurally diverse type II RAF inhibitors (belvarafenib, tovorafenib, and naporafenib) exhibit relative sparing of ARAF (32). Thus, differences in the conformational adaptability upon inhibitor binding to ARAF as compared to BRAF and CRAF, including propensity of the DFG-motif to reorient, could underlie its relative resistance to these drugs. This situation is reminiscent of the differential inhibition of Src kinase *versus* Abl by imatinib, a potent type II inhibitor of BCR-Abl. Imatinib is more than 1000-fold more potent against Abl than Src, which shares a highly conserved ATP site. An energetic penalty for adoption of the DFG-out conformation was initially proposed as a possible explanation for the lack of potency against Src (59), but subsequent work revealed equipotent inhibition of these two kinases by other type II inhibitors (60).

From a therapeutic perspective, the lack of potency against ARAF may be an advantage or a liability. ARAF is abundantly expressed across most human tissues, often together with other RAF family members (61). Blockade of all signaling through the RAS/RAF/MAP kinase cascade is poorly tolerated. Thus, for tumors that are driven by BRAF dimers, such as low-grade astrocytomas with KIAA2549:BRAF, sparing of ARAF may provide an improved therapeutic window relative to pan-RAF inhibition. A caveat with this view is that it remains unclear whether KIAA1549:BRAF can also signal *via* heterodimerization with other RAF isoforms, as has been demonstrated for oncogenic point mutants in BRAF (44). If this is the case, lack of ARAF inhibition may compromise efficacy in these tumors. Like tovorafenib and naporafenib, belvarafenib is a clinical-stage type II RAF inhibitor and is most potent against CRAF (32). While belvarafenib is reported to have similar potency against ARAF and BRAF in purified enzyme assays, it is less potent against ARAF in cells. Interestingly, point mutations in ARAF that confer resistance to belvarafenib have been identified both in model systems and in patients with BRAF^V600E^-driven melanoma treated with belvarafenib (32). Thus it is clear, at least in this context, that ARAF can mediate MAP kinase pathway signaling in the face of BRAF and CRAF inhibition. Further work will be required to better understand the sensitivity of ARAF to type II inhibitors more broadly and to understand the underlying basis for the differential sensitivity of these compounds across RAF isoforms.

## Experimental procedures

### Protein expression and purification

For assays, insect cells were coinfected with recombinant baculovirus expressing appropriate RAF and MEK constructs to generate the desired enzymatic complex. MEK1^SASA^:ARAF^SSDD^-14-3-3 ε was purified by infecting liter scale cultures of insect cells with separate recombinant baculovirus expressing either ARAF^274-606, G300S, Y301/302D^-14-3-3 ε with N-terminal His_6_ and StrepII tags (ARAF^SSDD^-14-3-3 ε) or full-length His_6_-MEK1^S218/222A^ (MEK1^SASA^). Cells were harvested approximately 72 h postinfection *via* centrifugation and lysed in Ni-binding buffer (pH 8.0, 50 mM Tris, 150 mM NaCl, 5 mM MgCl_2_, 1 mM tris(2-carboxyethyl)phosphine (TCEP), 1 μM AMP-PNP, and protease inhibitor cocktail from Thermo Fisher Scientific) *via* sonication. Clarified lysate was removed and bound to equilibrated Ni-NTA agarose beads (Qiagen) by gravity flow, washed with Ni-binding buffer supplemented with 30 mM imidazole, then eluted with Ni-binding buffer supplemented with 300 mM imidazole. Elutions containing expressed proteins were pooled and bound to an equilibrated StrepTrap HP column (GE Healthcare Life Sciences), washed with Ni-binding buffer, and then eluted using binding buffer supplemented with 5 mM desthiobiotin. Fractions were analyzed by SDS-PAGE and those containing the desired complex were pooled and concentrated to 1 ml by Amicon Ultra concentrator (30 MWCO, Millipore) before being injected onto a Superose 6 10/300 (GE Healthcare Life Sciences) column. SDS-PAGE analysis of the resulting fractions indicated that the coexpressed MEK1^SASA^ and ARAF^SSDD^-14-3-3 ε coeluted together along with some endogenous insect 14-3-3, forming a MEK1^SASA^:ARAF^SSDD^-14-3-3 ε RAF dimer complex that exhibits kinase activity in our TR-FRET assay.

MEK1^SASA^:BRAF^WT^:14-3-3 was isolated by first coinfecting liter scale cultures of insect cells with recombinant baculovirus expressing BRAF^419-766^ with an N-terminal His_6_ tag (BRAF^WT^) and with a separate virus, full-length MEK1^SASA^ described above. Approximately 65 to 72 h postinfection, cells were harvested by centrifugation and lysed in Ni-binding buffer (pH 8.0, 50 mM Tris, 150 mM NaCl, 10 mM MgCl_2_, 1 mM TCEP, 1 uM AMP-PNP, and protease inhibitor cocktail from Thermo Fisher Scientific) *via* sonication. Clarified lysate was removed and bound to equilibrated Ni-NTA agarose beads (Qiagen) by gravity flow, washed with Ni-binding buffer supplemented with 30 mM imidazole, and then eluted with Ni-binding buffer supplemented with 250 mM imidazole. Elutions containing expressed proteins were concentrated to 1 ml by Amicon Ultra concentrator (30 MWCO, Millipore) and injected onto a Superdex 200 Increase 10/300 column (GE Healthcare Life Sciences). SDS-PAGE analysis of the resulting fractions indicates that the coexpressed MEK1^SASA^ and BRAF^KD^ coelute together along with approximately stoichiometric amounts of endogenous insect 14-3-3, forming a MEK1^SASA^:BRAF^WT^:14-3-3 dimer complex that exhibits high levels of kinase activity in our TR-FRET assay. MEK1^SASA^:BRAF^V600E^ monomer complex was obtained by coinfecting insect cells with recombinant baculovirus expressing either full-length MEK1^SASA^ or BRAF^445-723, V600E^ (BRAF^V600E^) with an N-terminal His_6_ tag and a C-terminal intein tag. Similarly, to preparations for other complexes, cells were harvested approximately 72 h postinfection and lysed in lysis buffer (pH 8.0, 25 mM Tris, 100 mM NaCl, 5 mM MgCl_2_, 1 mM TCEP, 1 uM AMP-PNP, and protease inhibitor cocktail from Thermo Fisher Scientific). After ultracentrifugation at 40,000 rpm, clarified lysate was bound to equilibrated Ni-NTA agarose beads (Qiagen), washed with lysis buffer supplemented with 25 mM imidazole, and then eluted with lysis buffer supplemented with 500 mM imidazole. Elutions were pooled and incubated with 50 mM MESNA overnight, and then the next day applied to Chitin beads. The flowthrough was concentrated to 1 ml by Amicon Ultra concentrator (10 MWCO, Millipore) and then further purified by gel filtration using a Superdex 200 10/300 column (GE Healthcare Life Sciences). SDS-PAGE analysis of the resulting fractions indicated that MEK1^SASA^:BRAF^V600E^ eluted together as a monomer lacking 14-3-3 that exhibits high levels of kinase activity by TR-FRET.

To obtain MEK1:CRAF^WT^:14-3-3 dimers, insect cells were coinfected with recombinant baculovirus expressing either His_6_-MEK1^35-393^ (MEK1) with a C-terminal avi-tag or His_6_-CRAF^308-648^ (CRAF^WT^). MEK1:CRAF^WT^:14-3-3 was purified similarly to MEK1^SASA^:BRAF^WT^:14-3-3, using identical buffers. Briefly, after infection, cells were harvested, lysed, and ultracentrifuged. Clear lysate was bound to Ni-NTA agarose beads, washed, and eluted. After SDS-PAGE, elutions containing the complex were concentrated to 1 ml and injected onto a Superdex 200 10/300 column for further purification by gel filtration. These size-exclusion fractions were run on an SDS-PAGE gel, again revealing that MEK1 and CRAF^WT^ coelute with endogenous 14-3-3, and fractions corresponding to the MEK1:CRAF^WT^:14-3-3 dimer complex were pooled and concentrated for storage at −80 °C until used for assays. Our TR-FRET assay revealed that this complex was also active. MEK1^SASA^:CRAF^SSDD^:14-3-3 was isolated by coinfecting insect cells with the usual MEK1^SASA^-expressing baculovirus described above as well as His_6_-StrepII-CRAF^308-648, Y340/341D^ (CRAF^SSDD^)-expressing baculovirus. Like other complexes, cells were harvested, lysed, and ultracentrifuged and then bound, washed, and eluted from Ni-NTA agarose beads using the buffers described above for MEK1^SASA^:BRAF^WT^:14-3-3 purification. Elutions containing MEK1^SASA^, CRAF^SSDD^, and 14-3-3 were pooled and bound to an equilibrated prepacked StrepTrap HP column (GE Healthcare Life Sciences), washed with Ni-binding buffer, and then eluted using binding buffer supplemented with 5 mM desthiobiotin. Fractions were analyzed by SDS-PAGE, and elutions containing the complex were pooled and concentrated to 1 ml by Amicon Ultra concentrator (30 MWCO, Millipore) and then injected onto a Superdex 200 Increase 10/300 column. This complex also eluted for the column with endogenous, insect 14-3-3 proteins as an active MEK1^SASA^:CRAF^SSDD^:14-3-3 dimer.

Substrate for the TR-FRET assay was purified by first infecting insect cells with recombinant baculovirus expressing His_6_-MEK1^35-393^ (MEK1) with a C-terminal avi-tag to allow for biotinylation by birA. Approximately 3 days postinfection, cells are harvested *via* centrifugation, lysed, then ultracentrifuged at 40,000 rpm in Ni-binding buffer (pH 8.0, 50 mM Tris, 150 mM NaCl, 10 mM MgCl_2_, 2 mM TCEP, 1 uM AMP-PNP, and protease inhibitor cocktail from Thermo Fisher Scientific). Once clearly separated, the lysate supernatant was bound to Ni-NTA agarose beads, washed with Ni-binding buffer supplemented with 25 mM imidazole, then eluted with Ni elution buffer (pH 8.0, 50 mM Tris, 150 mM NaCl, 10 mM MgCl_2_, 2 mM TCEP, 1 uM AMP-PNP, and 250 mM imidazole). Ni fractions were analyzed by SDS-PAGE and relevant fractions were pooled then diluted with Ni binding buffer such that the imidazole concentration was 200 mM or less. This diluted volume was bound to MagStrep “type3” XT agarose beads (IBA Lifesciences) multiple times at less than 1 ml/min, washed with a small volume of Ni-binding buffer, then eluted using Strep elution buffer (100 mM HEPES, 150 mM NaCl, 10 mM MgCl_2_, 2 mM TCEP, 50 mM D+ biotin, pH 8.0). The concentration of protein in this elution was determined *via* Bradford assay, then ATP was added to a final concentration of 20 mM birA enzyme such that the ratio of MEK1 to birA was 50:1, and then incubated at 4 °C overnight. The birA used for biotinylation was expressed and purified in-house according to established procedures. Following biotinylation overnight, the elution volume was concentrated to 5 ml using an Amicon Ultra concentrator (30 MWCO, Millipore) and then further purified *via* size-exclusion chromatography on a Superdex 200 Increase 10/300 column. Substrate quality was evaluated by biotin labeling according to mass spectroscopy and comparison of TR-FRET activity profiles to in-house standards.

For crystallization purposes, residues 445 to 723 of the human BRAF kinase domain with fourteen solubilizing mutations (BRAF^14M^) were inserted into a bacterial expression vector with an N-terminal, Tobacco Etch Virus (TEV)-cleavable His_6_ tag. WT and BRAF^V600E^ constructs were prepared using the same protocol as previously described. Briefly, we expressed these constructs in BL21 DE3 *E. coli* at 37 °C with 180 rpm agitation until optical density at 600 nm (A_600_) reached 0.5 to 0.7, at which point the flasks were cooled by immersion in ice water for 10 to 15 min. Expression was induced by the addition of 0.5 mM IPTG and overnight incubation at 18 °C with agitation.

Cells were harvested *via* centrifugation and were resuspended in roughly 20 ml of Bind/Wash buffer (50 mM Tris pH 7.0, 250 mM sodium chloride, 30 mM imidazole, and 1 mM TCEP) per liter of culture with 1 mM PMSF to inhibit serine protease activity. Cells were lysed *via* sonication. All proceeding steps were done on ice. The lysate was clarified by centrifugation for 2 h at 17,000*g*. After centrifugation, the cleared lysate was filtered using 0.8 μm filters. The cleared lysate was then applied to a pre-equilibrated 5 ml bed volume HisTrap column. The column was washed with 10 column volumes Bind/Wash buffer on an FPLC, and the BRAF protein eluted using a 100 ml gradient from 0 to 50% elution buffer (50 mM Tris pH 7.0, 250 mM NaCl, 250 mM imidazole, and 1 mM TCEP). The peak containing BRAF eluted between 25 to 32% elution buffer. Fractions were pooled and the His_6_ tag cleaved by the addition of TEV protease and incubation overnight at 4 °C. Following cleavage, the BRAF/TEV mixture was concentrated using a 30,000 NMWCO Amicon spin concentrator to ≤15 mg/ml and injected onto a GE S75 10/300 size-exclusion column pre-equilibrated with 1.5 column volumes of sizing buffer (Tris pH 7.0, 250 mM sodium chloride, 1 mM TCEP). The resulting fractions were analyzed by SDS-PAGE, and fractions containing BRAF were pooled and concentrated to 20 to 25 mg/ml and flash frozen. Final yield for the WT and V600E preparations were approximately 15 and 10 mg/liter of culture, respectively.

### Crystallization and structure determination

Tovorafenib complex crystals were prepared by diluting BRAF protein to 10 to 12 mg/ml using sizing buffer prior to the addition of 200 nM tovorafenib and 5 mM MgCl_2_. Urchin-like crystal clusters were obtained in 150 nl sitting drops mixed 1:1 with a reservoir solution containing 8% Tacsimate, pH 8, and 20% PEG 3350. Crystals were optimized using the Hampton Additive Screen, and B9 (sodium thiocyanate) and F9 (benzamidine HCl) appeared to improve crystal quality. These conditions were further optimized in 2 μl hanging drops over wells containing 16 to 26% PEG 3350 and either sodium thiocyanate or benzamidine HCl. After optimization, the thiocyanate condition yielded rhomboidal crystals between 50 to 80 μm across, and the benzamidine condition yielded large plates between 100 to 300 μm across. Naporafenib complex crystals were prepared similarly to tovorafenib, substituting naporafenib for tovorafenib. Initial crystals were obtained in reservoir solutions of 0.2 M MgCl_2_, 0.1 M tris pH 8.5, and 25% PEG 3350 and 8% Tacsimate, pH 8, and 20% PEG 3350. These conditions were optimized over wells of pH 6, 7, and 8. Crystals were harvested and flash frozen in liquid nitrogen with 20% glycerol as a cryoprotectant. X-ray diffraction data were collected at 100 K using NE-CAT beamline ID-24-C at the Advance Photon Source, Argonne National Laboratory, at a wavelength of 0.9786 A. Data were integrated and merged using XDS and scaled using Aimless in the CCP4 suite or by the Xia2 suite using the Dials mode (62, 63, 64, 65). Crystals from the thiocyanate condition diffracted to 3.5 A. The structure was phased by molecular replacement in PHASER using a previously determined structure of the BRAF kinase–bound naporafenib as an initial search model (PDB 6N0P) (36). Inhibitors were modeled into positive F_o_-F_c_ density refined using PHENIX.REFINE coupled with successive rounds of manual model building in COOT (66, 67). The resulting structures have been deposited in the Protein Data Bank with the accession codes 6V34 (BRAF^V600E^-tovorafenib), 8F7O (WT BRAF-tovorafenib), and 8F7P (WT BRAF-naporafenib).

### Kinase inhibition assays

Inhibition assays were performed using a modified HTRF KinEASE tyrosine kinase assay kit (Cisbio). Rather than the provided kit substrate, we purified MEK1^35-393^ and biotinylated it (MEK-B) in-house using birA enzyme. Inhibitors were dispensed into black 384-well plates using an HP300e dispenser and normalized to 1% final DMSO concentration per well. Kit assay buffer was supplemented with purified RAF at a final concentration of 1 nM for MEK1^SASA^:BRAF^KD^:14-3-3 and MEK1^SASA^:CRAF^SSDD^:14-3-3, 4 nM for MEK1:CRAF^KD^:14-3-3, and 10 nM for MEK1^SASA^:ARAF^SSDD^-14-3-3 ε, as well as purified biotinylated MEK-B at a final concentration of 250 nM. Supplemented kinase buffer was dispensed into 384-well plates using a Multidrop combi dispenser and incubated with inhibitors at room temperature for 40 min before reactions were initiated by 200 uM ATP dispensed using the Multidrop combi dispenser. Plates were quenched after 30 min at room temperature using the kit detection buffer supplemented with XL665 and PAb Anti-phospho MEK1/2-Eu (Cisbio). The FRET signal ratio was measured at 665 and 620 nm using a PHERAstar microplate reader and processed using GraphPad Prism fit to a three-parameter dose-response model with Hill Slope constrained to −1 and a four-parameter dose-response model that fits the Hill Slope to the data. Assays were performed in triplicate three independent times.

## Data availability

Protein structures have been deposited into the Protein Data Bank with the codes 6V34, 8F7O, and 8F7P, all other data are included in the manuscript.

## Supporting information

This article contains supporting information.

## Conflict of interest

M. J. E. is a consultant for Novartis and receives sponsored research support from Novartis Institutes for Biomedical Research and Springworks Therapeutics. All other authors declare that they have no conflicts of interest with the contents of this article.
