# Supplementary material for: Structure and RAF family kinase isoform selectivity of type II RAF inhibitors tovorafenib and naporafenib
Source: J Biol Chem. 2023 Mar 22;299(5):104634. doi: 10.1016/j.jbc.2023.104634 (PMC10149214; doi:10.1016/j.jbc.2023.104634)
Supplement: Supporting information [file mmc1.docx]

**Structure and RAF isoform selectivity of Type II RAF inhibitors tovorafenib and naporafenib**

Emre Tkacik^1,2,*^, Kunhua Li^1,2,3,*^, Gonzalo Gonzalez-Del Pino^1,2,4^, Byung Hak Ha^1,2^, Javier Vinals^1,2^, Eunyoung Park^1,2,5^, Tyler Beyett^1,2^, and Michael J. Eck^1,2^

Affiliations:

^1^Department of Cancer Biology, Dana-Farber Cancer Institute, Boston, MA 02215, USA.

^2^Department of Biological Chemistry and Molecular Pharmacology, Harvard Medical School, Boston, MA 02115, USA.

^3^Present address: FOG Pharmaceuticals Inc, 30 Acorn Park Dr, Cambridge, MA 02140, kunhuali@gmail.com

^4^Present address: Tufts University School of Medicine, 136 Harrison Avenue, Boston, MA 02111, gonzalo.gonzalez_del_pino@tufts.edu

^5^Present address: Pfizer Boulder Research & Development, 1825 33^rd^ St., Boulder, CO 80301, cryoman99@gmail.com

*These authors contributed equally.

**List of Supporting Information:**

Table S1. Data collection and refinement statistics.

Figure S1. Crystal structure of tovorafenib with BRAF^WT^.

Figure S2. Tovorafenib and naporafenib induce a change domain orientation.

Figure S3. Re-examination of prior BRAF structures reveals domain-swap.

**Table S1. Data collection and refinement statistics.***

|  | **Tovorafenib**  **BRAF^WT^** | **Tovorafenib BRAF^V600E^** | **Naporafenib BRAF^WT^** |
| --- | --- | --- | --- |
| PDB Accession | 87FO | 6V34 | 87FP |
| Resolution range | 47.64 - 3.54  (3.67 - 3.54) | 24.87 - 3.15  (3.26 - 3.15) | 46.75 - 2.74  (2.84 - 2.74) |
| Space group | P2_1_2_1_2 | P2_1_2_1_2_1_ | P2_1_ |
| Unit cell  *a, b, c* (Å)  a, b, 𝛾 (°) | 57.27, 85.83, 121.02  90, 90, 90 | 48.80, 85.24, 122.91  90, 90, 90 | 49.65, 101.75, 58.69  90, 109.7, 90 |
| Total reflections | 94831 (9739) | 55846 (5306) | 83309 (8365) |
| Unique reflections | 7567 (732) | 9188 (892) | 14435 (1433) |
| Multiplicity | 12.5 (13.3) | 6.1 (5.9) | 5.8 (5.8) |
| Completeness (%) | 98.01 (97.98) | 96.91 (96.96) | 99.26 (99.30) |
| Mean I/sigma(I) | 3.99 (1.12) | 7.56 (1.55) | 7.88 (1.91) |
| Wilson B-factor | 66.97 | 75.06 | 50.15 |
| R_merge_ | 0.455 (2.005) | 0.2515 (1.565) | 0.1714 (1.023) |
| R_meas_ | 0.475 (2.084) | 0.2765 (1.716) | 0.189 (1.124) |
| R_pim_ | 0.1336 (0.563) | 0.1126 (0.6898) | 0.07824 (0.458) |
| CC1/2 | 0.972 (0.55) | 0.989 (0.7) | 0.993 (0.819) |
| Reflections used in refinement | 7551 (729) | 9066 (892) | 14391 (1428) |
| Reflections used for R-free | 344 (25) | 483 (37) | 687 (79) |
| R_work_ | 0.2427 (0.3030) | 0.2612 (0.3735) | 0.2162 (0.3183) |
| R_free_ | 0.2864 (0.3440) | 0.2878 (0.3636) | 0.2625 (0.4272) |
| Number of non-hydrogen atoms | 4256 | 4087 | 4271 |
| protein | 4192 | 4023 | 4176 |
| ligands | 64 | 64 | 72 |
| solvent | 0 | 0 | 23 |
| Protein residues | 526 | 503 | 520 |
| RMS bonds (Å) | 0.003 | 0.003 | 0.004 |
| RMS angles (°) | 0.59 | 0.91 | 0.68 |
| Ramachandran  favored (%) | 93.22 | 94.25 | 95.67 |
| allowed (%) | 6.78 | 5.75 | 4.13 |
| outliers (%) | 0.00 | 0.00 | 0.20 |
| Rotamer outliers (%) | 3.92 | 0.00 | 8.53 |
| Clashscore | 8.39 | 4.30 | 5.66 |
| Average B-factor | 70.68 | 81.50 | 47.94 |
| macromolecules | 70.77 | 81.73 | 47.99 |
| ligands | 64.38 | 66.90 | 46.85 |
| solvent |  |  | 43.11 |

*Statistics for the highest-resolution shell are shown in parentheses.

| 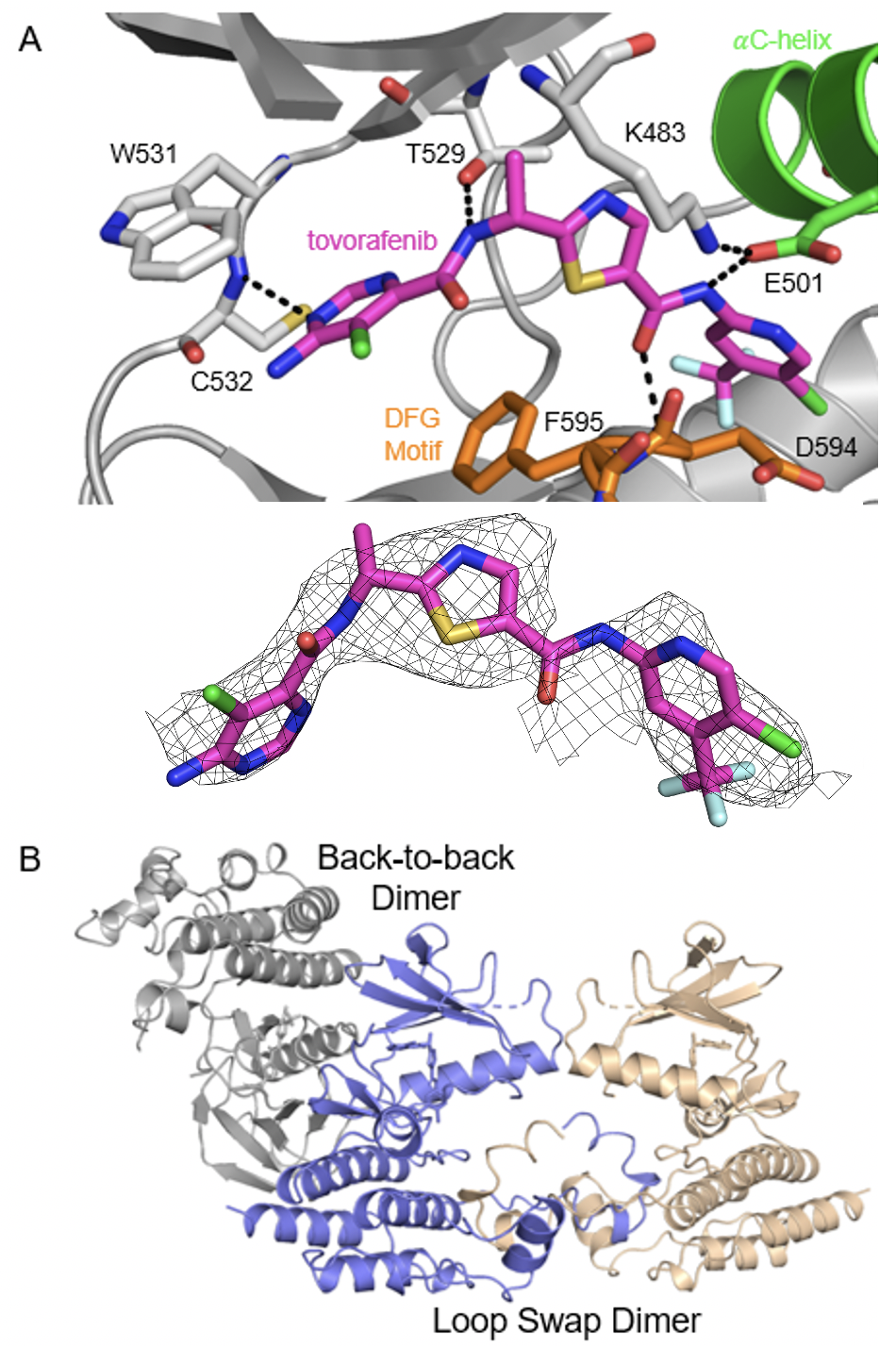 |
| --- |
| **Figure S1. Crystal structure of tovorafenib with BRAF^WT^.** A, Binding mode and inhibitor density for tovorafenib. The 2F_o_-F_c_ simulated annealing composite omit electron density map for tovorafenib in complex with BRAF contoured at 1σ. B, A domain-swap in the region of the kinase activation loop results in formation of a “face-to-face” dimer in the BRAF^WT^ crystals. The subdomain exchange occurs in one of the two protomers of the back-to-back BRAF dimer that composes the asymmetric unit. |

| **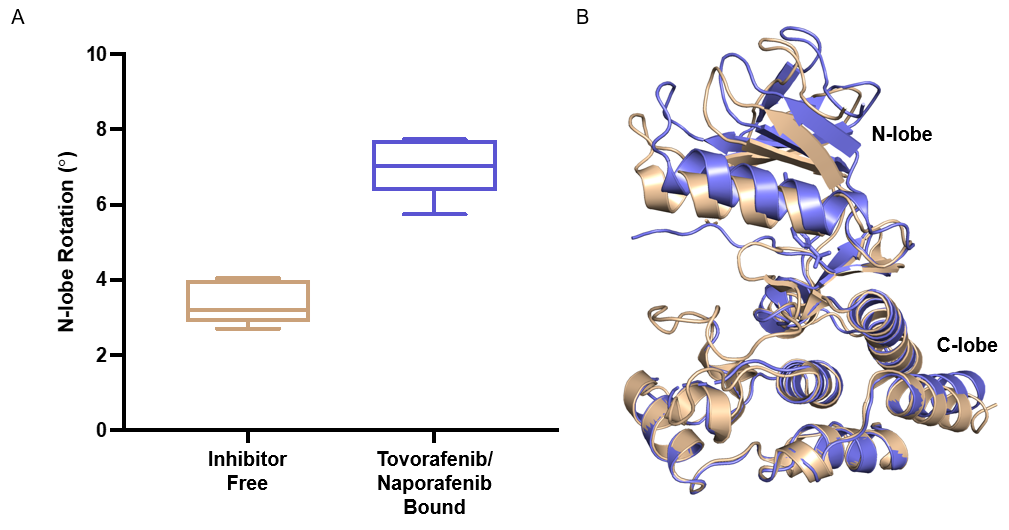** |
| --- |
| **Figure S2. Tovorafenib and naporafenib induce a small change in the relative orientations of the N- and C-lobes of the BRAF kinase domain.** A, Box and whisker plot showing change in relative N/C lobe orientations in five inhibitor-free BRAF molecules from two crystal structures (PDB IDs 4MNE and 6XAG) compared with that in the three tovorafenib or naporafenib-bound structures described here. Changes in the domain opening angle (°) were measured by first superimposing the kinase C-lobes, and then calculating the rotation needed to bring the N-lobes into alignment. Values reported are relative to chain B of PDB entry 4MNE, the most “closed” of the inhibitor-free structures. B, Illustration of the domain opening induced by naporafenib. When the BRAF^WT^/naporafenib structure (blue) and chain B of 4MNE (cream) are superimposed based on their C-lobes, a ~7° rotation is required to bring their N-lobes into register. |


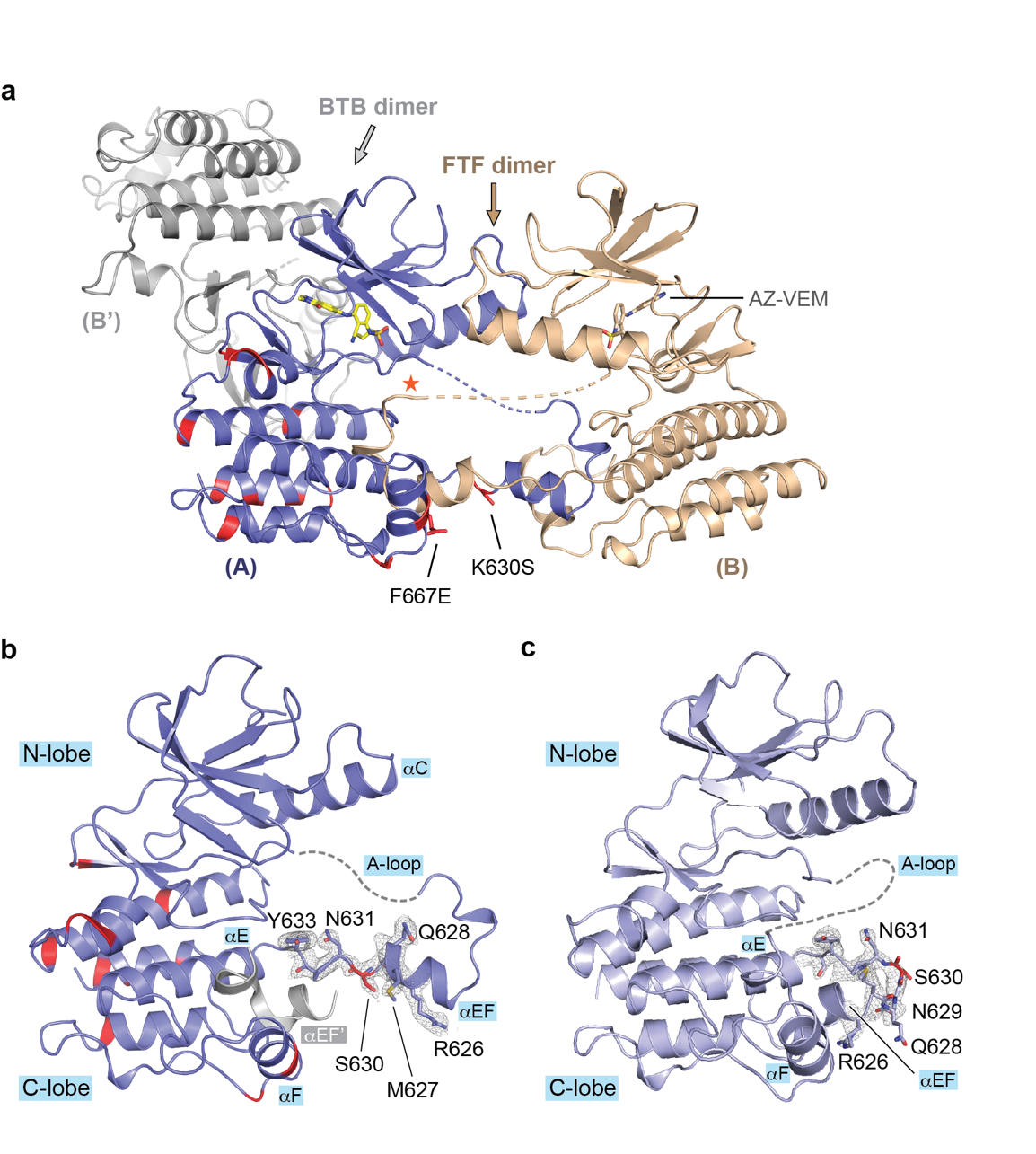


**Figure S3. Re-examination of prior BRAF structures reveals the presence of a domain-swap in the BRAF kinase domain.** A, PDB entry 5ITA contains a domain-swapped BRAF kinase that forms both back-to-back dimer and face-to-face dimer. Disordered partial activation loops (residue 598-613 in chain A) are shown as dashed lines. Methods: Inspection of electron density maps for PDB entry 5ITA revealed density corresponding to the swapped activation segment region. Accordingly the model was rebuilt in this region and re-refined to an R_work_/R_free_ of 19.0/23.3 at 1.95Å resolution. Similar results were obtained with re-refinement of PDB entries 4XV2 and 4CQE. B, Detailed view of domain-swapped activation loop and αEF regions in re-refined PDB Entry 5ITA. A 2F_o_-F_c_ electron density map, contoured at 1.0 σ, is shown for a key portion of the swapped region. The αEF’ region, as originally modeling in 5ITA is shown in light grey. Of note, residues 627-630 were not modeled in the original structure. In both panels A and B, engineered surface mutations of BRAF^16mut^, including K630S in the region of the subdomain swap, are colored in red. C, BRAF^V600E^ in complex with PLX4032 (PDB entry 3OG7) shares the similar face-to-face interface with the domain-swapped BRAF structures, but is not domain-swapped.
